# Utility of Zebrafish Models of Acquired and Inherited Long QT Syndrome

**DOI:** 10.3389/fphys.2020.624129

**Published:** 2021-01-15

**Authors:** Kyle E. Simpson, Ravichandra Venkateshappa, Zhao Kai Pang, Shoaib Faizi, Glen F. Tibbits, Tom W. Claydon

**Affiliations:** ^1^Molecular Cardiac Physiology Group, Department of Biomedical Physiology and Kinesiology, Simon Fraser University, Burnaby, BC, Canada; ^2^Department of Cardiovascular Science, British Columbia Children’s Hospital, Vancouver, BC, Canada

**Keywords:** Long-QT Syndrome, zebrafish, cardiac electrophysiology, inherited LQTS, acquired LQTS, CRISPR, hERG, zERG

## Abstract

Long-QT Syndrome (LQTS) is a cardiac electrical disorder, distinguished by irregular heart rates and sudden death. Accounting for ∼40% of cases, LQTS Type 2 (LQTS2), is caused by defects in the Kv11.1 (hERG) potassium channel that is critical for cardiac repolarization. Drug block of hERG channels or dysfunctional channel variants can result in acquired or inherited LQTS2, respectively, which are typified by delayed repolarization and predisposition to lethal arrhythmia. As such, there is significant interest in clear identification of drugs and channel variants that produce clinically meaningful perturbation of hERG channel function. While toxicological screening of hERG channels, and phenotypic assessment of inherited channel variants in heterologous systems is now commonplace, affordable, efficient, and insightful whole organ models for acquired and inherited LQTS2 are lacking. Recent work has shown that zebrafish provide a viable *in vivo* or whole organ model of cardiac electrophysiology. Characterization of cardiac ion currents and toxicological screening work in intact embryos, as well as adult whole hearts, has demonstrated the utility of the zebrafish model to contribute to the development of therapeutics that lack hERG-blocking off-target effects. Moreover, forward and reverse genetic approaches show zebrafish as a tractable model in which LQTS2 can be studied. With the development of new tools and technologies, zebrafish lines carrying precise channel variants associated with LQTS2 have recently begun to be generated and explored. In this review, we discuss the present knowledge and questions raised related to the use of zebrafish as models of acquired and inherited LQTS2. We focus discussion, in particular, on developments in precise gene-editing approaches in zebrafish to create whole heart inherited LQTS2 models and evidence that zebrafish hearts can be used to study arrhythmogenicity and to identify potential anti-arrhythmic compounds.

## Long-QT Syndrome and hERG Channels

Long QT syndrome (LQTS) is characterized by prolongation of the heart rate-corrected QT interval (QTc) and dysmorphic T-waves on surface electrocardiogram (ECG) recordings, and increases risk of cardiac arrhythmia (Alders et al., 1993; Roden, 2008; Schwartz et al., 2012). QTc prolongation occurs as a result of aberration in one of several cardiac ion channels resulting in anomalous depolarization or repolarization of cardiomyocytes and prolongation of the action potential duration (APD). APD prolongation that increases the QTc above the 95th percentile of the normal range (350–450 ms) is used in the risk stratification of LQTS (Postema and Wilde, 2014). LQTS can be acquired or congenital, the latter accounting for approximately 1 in 2500 people, with the former being more prevalent and attributable to electrolyte imbalances or adverse drug effects (Schwartz et al., 2009). Inherited LQTS has been linked to mutations in several cardiac ion channels with *KCNQ1* (LQTS1), *KCNH2* (LQTS2), and *SCN5A* (LQTS3) being the most common LQTS genes, accounting for ∼90% of all genotype-positive cases (Schwartz et al., 2012). Altered ionic currents in these cases prolongs the APD, which increases the susceptibility to early after depolarizations (EADs) and triggered activity and also creates dispersion of repolarization across the ventricular wall creating a substrate for arrhythmias (El-Sherif et al., 2019). A prolonged QT interval predisposes individuals to a form of ventricular tachycardia, torsade de pointes (TdP), which can degenerate to ventricular fibrillation and syncope, cardiac arrest, and sudden death (Alders et al., 1993; Schwartz et al., 2012).

Variants in the *KCNH2* gene that cause channel dysfunction (loss of trafficking or gating changes that reduce the time channels spend in the open state) are linked to LQTS2, which accounts for ∼40% of LQTS cases (Schwartz et al., 2012). More than 500 *KCNH2* gene mutations associated with LQTS2 have been identified (Alders et al., 1993; Schwartz et al., 2009; Shah et al., 2019). *KCNH2* encodes the α-subunit Kv11.1 (also known as the human Ether-à-go-go-Related Gene, hERG), which is responsible for the major repolarizing current, I_*Kr*_, during phase 3 of the cardiac action potential (Shah and Carter, 2008). As such, hERG channels are important for physiological suppression of EADs and triggered activity (Smith et al., 1996). Reduced hERG currents due to genetic variants prolongs repolarization, increasing the susceptibility to triggering cardiac events such as EADs, which can lead to lethal arrhythmias and sudden death. Acquired forms of LQTS, thought to be more prevalent than the inherited form, are mostly due to adverse drug effects or electrolyte imbalance and are almost exclusively associated with blocking of hERG channels and reduced repolarization.

Drug-induced cardiotoxicity as a result of hERG channel block is one of the major hurdles in drug discovery and development and has been a primary reason for the withdrawal of many clinically approved drugs from the market (Kannankeril et al., 2010; Mandenius et al., 2011). hERG assays using mammalian heterologous expression systems (e.g., HEK-293, CHO cells) have been the gold standard in predicting cardiotoxicity. However, translatability of this assay is limited due to the lack of complexity of ion currents that are expressed in native cardiomyocytes, which may lead to the loss of potential drug candidates in the early stage of drug discovery (Liang et al., 2013). As such, there is significant interest in the generation of screening platforms that use more complex physiologically relevant cell or animal models. Furthermore, there is a need to better understand the complexity of genotype-phenotype correlations and the underlying fundamental mechanisms of inherited LQTS to be better able to risk-stratify variants and to develop and test effective targeted therapeutics. Thus high-throughput translational models that enable study of the complex mechanisms of cardiac repolarization and its alterations in congenital and acquired LQTS are highly sought after. In recent years, the use of zebrafish to provide such a model has gained traction (van Opbergen et al., 2018; Tanaka et al., 2019). Here we briefly discuss the utility of zebrafish as a cardiac model and then review the use of zebrafish as a model of acquired and inherited LQTS2.

## Zebrafish as a Cardiac Model

### Morphological Characteristics

The zebrafish heart is distinct from mammalian hearts, most clearly by the presence of two, rather than four, cardiac chambers. However, even with only one atrium and one ventricle, the zebrafish heart is remarkably mammalian-like in a number of ways that suit its use as a model system. The zebrafish heart develops, starting as a single conducting tube, at just 24 h post-fertilization (hpf), and over 72 h, develops nodal activity, separation of atrium and ventricle, and coordinated inter-chamber conduction (Chi et al., 2008). This rapid timeline of development allows for observation of a functional heart early in development, proving advantageous for studying, for example, toxicological screens, modifying mutations, and developmental pathways (Bakkers, 2011; Sarmah and Marrs, 2016). These investigations are greatly aided by the transparency of zebrafish larvae, which enable direct visualization of cardiac function using simple motion capture, or genetically encoded indicators, to monitor outcome measures such as bradycardia, tachycardia, or 2:1 atrioventricular block (Garrity et al., 2002; Chan et al., 2009). Furthermore, the diminutive size of the zebrafish larvae permits oxygen exchange via passive diffusion, ameliorating the need for a functional cardiac pump and this allows for study of potentially severe cardiac defects that might otherwise induce mortality in other model systems (Kang et al., 2018).

### Electrical Properties

The sinoatrial node (SA node) is a heterogeneous cluster of cells that forms the pacemaker region, responsible for the initiation of cardiac depolarization, and chronotropic responses of the heart. In zebrafish the SA node has been shown to develop very early, with the more primitive “heart tube” showing a constant and linear conduction pathway at 24 hpf. Pacemaking activity was shown to be critical in zebrafish heart rate regulation by the homozygous *slow mo* zebrafish variant, which resulted in attenuated pacemaking in isolated cardiac myocytes (Baker et al., 1997; Warren et al., 2001). Using a genetically encoded GFP linked to a transcription factor (*Isl1*) previously identified in mammalian SA nodal progenitor cells, the zebrafish SA node was identified as a ring of tissue at the junction of the sinus venosus and atrium (Tessadori et al., 2012). Isolation and patch clamp electrophysiological assessment of GFP+ cells revealed that these cells produce spontaneous action potentials, demonstrating their role in pacemaker activity (Tessadori et al., 2012). Subsequent work using transgenic zebrafish lines expressing GFP in conducting tissue combined with *hcn4* and *shox2* nodal-specific markers confirmed the presence of conducting cells in the SA node region of the zebrafish heart (Poon et al., 2016). These studies also identified dense innervation around the GFP-labeled cells, consistent with autonomic nervous system chronotropic regulation of the pacemaker site (Poon et al., 2016).

At approximately 2 days post-fertilization (dpf), a canal of tissue separates the zebrafish atrium and ventricle. This tissue appears to function similarly to the mammalian atrioventricular node (A-V node), in that it delays electrical propagation between the atrium and ventricle, allowing for coordinated contraction of the two chambers (Sedmera et al., 2003; Milan et al., 2006a; Chi et al., 2008). Using optical mapping approaches, Sedmera et al. (2003), first identified a slowing of current through the junction between atrium and ventricle. A subsequent study mapped action potential morphology in the atrioventricular canal and demonstrated the presence of an action potential configuration that was distinct from that observed in either atrium or ventricle, and that contained a slow diastolic depolarization phase consistent with mammalian atrioventricular electrical activity (Chi et al., 2008). Interestingly, Stoyek et al. (2016), found that the atrioventricular canal functioned as a secondary site of pacemaker activity; upon vagal nerve stimulation, the source of spontaneous depolarization shifted from the SA node to the A-V node as is observed in mammalian hearts upon vagal stimulation.

Zebrafish hearts function as a syncytium with cell-to-cell communication afforded by similar connexin proteins to those in mammals, with orthologs of Cx40, Cx43, and Cx45 (Cheng et al., 2004; Christie et al., 2004; Chi et al., 2010). Christie et al. (2004), identified and characterized zfCx45.6 showing that it possessed 63% sequence identity with human Cx40 (Verheule and Kaese, 2013). Functional assessment of zfCx45.6 in dual voltage clamp *Xenopus* oocytes demonstrated functional gap junction formation with similar conductance and voltage-dependence to mammalian Cx40 (Christie et al., 2004). Knockdown or mutated variants (*dco^*s*226^*) of zfCx48.5 (also described as zfCx46) produced uncoordinated contractions and decreased cardiac output (Cheng et al., 2004; Chi et al., 2010) indicative of a role in gap junction formation in the zebrafish heart. zfCx43 is the zebrafish ortholog to mammalian Cx43 showing 71% sequence identity with human Cx43 and expresses in the developing heart in a similar pattern to the mouse (Chatterjee et al., 2005; Iovine et al., 2005). The Cx45 ortholog, zfCx43.4, has high identity with human Cx45 (Essner et al., 1996), but no data have localized zfCx43.4 to the heart, and its function may differ from mammalian Cx45 (Barrio et al., 1997; Desplantez et al., 2003).

In terms of whole organ electrical propagation, recordings of ECG in larval and adult zebrafish demonstrate similar temporal sequence of activation and relaxation to that observed in human hearts (Milan et al., 2006b). Recently, ECG and high resolution optical detection of temporal voltage changes within the ventricle during activation and relaxation were used to correlate ECG waveforms with voltage gradients in the adult zebrafish heart (Zhao et al., 2020). These studies suggested that zebrafish hearts rely on epicardial gradients more strongly than transmural gradients as in humans, perhaps as a result of the differences in myocardium thickness of the ventricular wall, and this may have implications for translation of findings related to arrhythmia initiation and maintenance in zebrafish to the human (Zhao et al., 2020).

### Cardiac Electrophysiology

The zebrafish ventricular action potential is remarkably similar to that in humans, more so than other small mammalian systems, such as the murine model. Most noticeably, the significant plateau in phase 2 is pronounced in zebrafish ventricular myocytes as it is in the human, and aside from the rapid repolarization observed in epicardial regions during phase 1, all other action potential phases are shared (Vornanen and Hassinen, 2016). Direct comparison of action potentials recorded from adult zebrafish ventricular cells with those from human papillary muscle and murine ventricular strips revealed the closely associated morphology of zebrafish and human ventricular action potential (Nemtsas et al., 2010). As a result, measurements of APD in adult and larval (e.g., 3 dpf) zebrafish hearts report values of ∼140–230 ms (see Table 1), which are remarkably dependent on temperature (Rayani et al., 2018), but reflect the duration of the human ventricular action potential reasonably well (Alday, 2014; Vornanen and Hassinen, 2016; Hull et al., 2019; Shi et al., 2020; Zhao et al., 2020). The QT interval measured from ECG recordings in zebrafish demonstrates comparable resting heart rate and conduction intervals with humans and the QT interval has a near linear relationship with the RR interval (Milan et al., 2006b), features that are imperative for a model examining LQTS that involves delayed ventricular repolarization and prolongation of the APD and QTc interval.

**TABLE 1:** Acquired LQTS2-associated drug effects on cardiac electrical activity in zebrafish larvae and adults.

	Larval zebrafish hearts		
Drug	Heart rate	A-V dissociation	APD
100 compounds	Bradycardia with 36 (22 known to increase QT and TdP)^5^		
11 hERG blockers	Bradycardia with all^6^	2:1 block with all (at higher concentrations)^6^	
9 QT-prolonging compounds	Bradycardia with all^10^	2:1 block with all (*R*^2^ = 0.93; IC_50_ and 2:1 block)^10^	
35 compounds		2:1 block with 14 of 17 known to prolong QT^9^	
**Amiodarone**			
20 μg/ml	Bradycardia (HR reduced by 30%)^18^		
**Astemizole**			
60 μg/ml	Bradycardia (HR reduced by 50%)^18^	2:1 block^9^	
1 μM		Heart failure^9^	
5 μM			
**Cisapride**			
5 μM		2:1 block^9^	
10 μM		Ventricular failure^9^	
20 μM		Heart failure^9^	
**Dofetilide**			
10 nM		2:1 block^1^	Increased by 64 ± 45 ms (+28%)^1^
12 μM			
**Haloperidol**			
5 μM		2:1 block^9^	
10 μM		Ventricular failure^9^	
50 μM		Heart failure^9^	
**Tamoxifen**			
20 μM	Bradycardia (HR reduced by 75%)^18^		
**Terfenadine**			
Unknown	Bradycardia^9^	2:1 block^2,8^	Increased from 231 ± 5 to 245 ± 7 ms (100 nM; 6%)^3^
100 nM		2:1 block^9^	Increased by 58 ± 15% (100 nM)^2^
1 μM		Rescue of *reggae* phenotype in 54% of larvae^7^	
5 μM		Heart failure (20 μM)^9^	
10 μM		2:1 block^7^	
20 μM			
25 μM			
**2-MMB**			
50 μM		Suppressed 2:1 block in *breakdance*^4^	Decreased APD in *breakdance* from 570 ± 23 to 376 ± 66 ms (−34%)^4^
**Flurandrenolide**			
50 μM		Suppressed 2:1 block in *breakdance*^4^	Decreased APD in *breakdance* from 482 ± 83 to 338 ± 44 ms (−30%)^4^
**hERG blockers**				
**Astemizole**				
50 μM	Decreased from 308 ± 77 ms by −16 ± 6%^11^		Increased from 242 ± 54 ms by +18 ± 9%^11^	
**Dofetilide**				
50 nM		APD_75_ increased from 159 ± 8 to 193 ± 9 ms (+21%)^14^		Increased triangulation (APD_75_-APD_25_) from 75 to 89 ms (+16%)^14^
100 nM		APD_90_ increased by 75 ms^15^		Increased APD^90^ and Peak-Peak variance^17^
10 μM				
**E-4031**				
1 μM	Bradycardia^12^	APD_90_ increased from 144 10 to 179 9 ms (+20%)^13^	QTc increased from 439 ± 39 to 529 ± 27 ms	Increased triangulation (APD_90_-APD_30_) from 10 to 16 ms (+60%)^13^
10 μM		Increased	(+21%)^12^	Spontaneous EADs (1 s^–1^)^16^
20 + 1 μM Isoproterenol		APD_90_ increased to >3000 ms^16^		
**Haloperidol**				
100 μM	Decreased from 308 ± 77 ms by −38 ± 14%^11^		Increased from 242 ± 54 ms by +16 ± 11%^11^	
**Pimozide**				
10 μM	NSD from 308 ± 77 ms^11^		Increased from 242 ± 54 ms by +17 ± 9%^11^	
**Terfenadine**				
200 nM 100 μM	NSD from 308 ± 77 ms^11^	APD_75_ increased from 183 ± 7 to 216 ± 8 ms (+17%)^14^	Increased from 242 ± 54 ms by +11 ± 6%^11^	Increased triangulation (APD_75_-APD_25_) from 92 to 94 ms (+2%)^14^
**hERG activators**				
**NS1643**				
20 μM		APD_75_ NSD from 209 ± 13^14^		Increased triangulation (APD_75_-APD_25_) from 83 to 101 ms (+18%)^14^
		APD_25_ decreased from 126 ± 6 to 101 ± 6 ms (−19%)^14^		
**PD-118057**				
40 μM		APD_75_ decreased from 193 ± 6 to 176 ± 6 ms (−8%)^14^		Decreased triangulation (APD_75_-APD_25_) from 82 to 69 ms (−16%)^14^
**RPR260243**				
10 μM		APD_75_ decreased from 196 ± 9 to 164 ± 7 ms (−16%)^14^		Decreased APD_90_ and Peak-Peak variance in dofetilide treated hearts^17^
30 μM		APD_90_ decreased from 132 ± 4 to 111 ± 2 ms (−16%)^17^		Decreased triangulation (APD_75_-APD_25_) from 95 to 71 ms (−25%)^14^
				Decreased triangulation (APD_90_-APD_25_) from 74 ± 3 to 45 ± 3 ms (−39%)^17^
				Increased max slope of ERC from 0.69 ± 0.08 to 1.36 ± 0.26 (+97%)^17^
				Increased Post-Repolarization Refractory Period (PRRP) from 58 ± 4 to 75 ± 7 ms (+23%)^17^

*NSD, not significantly different. Data from Milan et al., 2009^1^; Arnaout et al., 2007^2^; Alday, 2014^3^; Peal et al., 2011^4^; Milan et al., 2003^5^; Langheinrich et al., 2003^6^; Hassel et al., 2008^7^; Milan et al., 2006a^8^; Letamendia et al., 2012^9^; Mittelstadt et al., 2008^10^; Milan et al., 2006b^11^; Tsai et al., 2011^12^; Nemtsas et al., 2010^13^; Hull et al., 2019^14^; Genge et al., 2016^15^; Sacconi et al., 2020^16^; Shi et al., 2020^17^; Burns et al., 2005^18^.*

Building on earlier work characterizing zebrafish ventricular myocyte ion currents (Brette et al., 2008), Nemtsas et al. (2010), provided the most comprehensive description of cardiac currents responsible for the zebrafish ventricular action potential to date. Biophysical characterization and selective drug blockade under voltage clamp conditions, allowed these authors to examine Na^+^, Ca^2+^, and K^+^ in isolated zebrafish ventricular myocytes. These studies demonstrated key roles for voltage-gated Na^+^ channels in the action potential upstroke, and L-type and T-type Ca^2+^ channels in the maintenance of the plateau phase (Nemtsas et al., 2010; Zhang et al., 2010); the prominent role of the latter being somewhat different from that in the adult mammalian ventricle (Haverinen et al., 2018). Other studies suggest a prominent role for Na^+^-Ca^2+^ exchange current at depolarized voltages during the plateau phase (Zhang et al., 2010). Dependence of the ventricular resting membrane potential on I_*K*__1_ expression was also demonstrated (Nemtsas et al., 2010), although the molecular correlate appears to be a different inward rectifier subfamily member in zebrafish and humans (Hassinen et al., 2015; Vornanen and Hassinen, 2016). Selective drug block initially indicated little functional presence of I_*Ks*_ during phase 3 repolarization in zebrafish (Nemtsas et al., 2010; Alday, 2014), however, expression of Kv7 K^+^ transcripts in cardiac tissue (Wu et al., 2014), and more recent studies demonstrate the presence of I_*Ks*_ current (Abramochkin et al., 2018). A consistent feature of zebrafish cardiac repolarization is the prominent role of I_*Kr*_ in phase 3 ventricular repolarization. I_*Kr*_ is conveyed by zERG channels from the *zkcnh6a* gene (Langheinrich et al., 2003; Scholz et al., 2009; Leong et al., 2010; Vornanen and Hassinen, 2016; Hull et al., 2019). The substantial genetic and pharmacological evidence for the importance of zERG channels and I_*Kr*_ as a critical driving force behind phase 3 repolarization suggests that zebrafish bear great potential as a model of both acquired and inherited LQTS as is discussed in more detail below.

## Zebrafish as a Model of Acquired Long-QT Syndrome

### HR, APD, and QT Interval as Markers of Arrhythmia in Zebrafish

Cardiotoxicity due to hERG channel blockade is a major hurdle in drug discovery and has resulted in high profile withdrawals from the market (Kannankeril et al., 2010; Mandenius et al., 2011). About 35–40% of drug candidates are dropped in the early development phase due to hERG toxicity issues, 19% due to cardiotoxicity, i.e., drug-induced arrhythmias (Mandenius et al., 2011). A wide variety of drugs (developed for cardiovascular or non-cardiovascular diseases) including antiarrhythmics, antipsychotics, antibiotics, antihistamines, amongst others are known to block hERG channels. Drugs that block hERG currents delay ventricular repolarization, which may lead to TdP and sudden death (Kannankeril et al., 2010). Mandatory guidelines require the testing of all early drug candidates for their hERG liability before filing an investigational new drug (IND) application (Hammond and Pollard, 2005; Lu et al., 2008). A hERG block assay is routinely used as a surrogate marker for QT prolongation (Gintant et al., 2016) and direct measurement of hERG currents using the patch-clamp technique has been the gold standard (Lawrence et al., 2005; Gintant et al., 2016). Non-cardiac mammalian CHO or HEK-293 cells, which artificially express hERG, are easy to maintain, can be sub-cultured for numerous passages, allow the formation of high resistance low noise seals, and lack contaminating currents (Kannankeril et al., 2010; Gintant et al., 2016); however, while effective in predicting the hERG block, they pose disadvantages in that these expression systems differ in their cellular environment, likely lack many important ancillary proteins for hERG channel modulation, and do not accurately produce the channels in their native form (McNeish, 2004; Kannankeril et al., 2010; Liang et al., 2013). As such, hERG assays using non-cardiac cells can produce false positive or false negative results, which may lead to the loss of potential drug candidates in the early stage of drug development (Liang et al., 2013). The use of primary cardiomyocytes offers an environment with naturally occurring subunit composition and other intracellular pathways or factors that may modulate channel properties (Kannankeril et al., 2010), but they are not proliferative and need to be isolated freshly for every experiment and since hERG currents are low in magnitude, and can be contaminated with other currents, recording currents accurately is challenging (McNeish, 2004; Kannankeril et al., 2010; Liang et al., 2013). More complex models, such wedge and isolated heart preparations, which use both electrophysiological and non-electrophysiological assays to test the proarrhythmic potential of the molecules during preclinical studies (Lawrence et al., 2005) are better at predicting APD prolongation and TdP risk, but lack throughput and are often costly alternatives. In recent years, zebrafish have emerged as a potential toxicological screening platform for compounds at risk of predisposing APD prolongation, TdP, and acquired LQTS in humans.

Zebrafish were introduced as a drug screening platform in the context of LQTS almost 20 years ago (Langheinrich et al., 2003; Milan et al., 2003; see Table 1). These studies recognized the advantages of using transparent 48–72 hpf larvae to monitor the effects of compounds on cardiac rhythmicity using relatively simple light microscopy imaging. Milan et al. (2003), tested the effects of 100 compounds on zebrafish heart rate. Of these, 36 caused bradycardia, and 21 of these are known to cause QT prolongation and TdP. Langheinrich et al. (2003), screened 11 diverse hERG blocking compounds in zebrafish and all 11 induced bradycardia, and 2:1 atrioventricular block at high concentrations, both of which are consequences of LQTS observed in humans (Motoike et al., 2000; Chang et al., 2004; Lee et al., 2006). These initial studies demonstrated that compounds which block hERG channels in heterologous cell assays and cause QT prolongation and TdP in humans have high affinity for the zERG ortholog in zebrafish. Rescue of the gain-of-function reggae zERG mutant cardiac phenotype by terfenadine block (Hassel et al., 2008) further demonstrated the targeted action of hERG-specific drugs in zebrafish. Indeed isolated zERG (*zkcnh6a*) channels have subsequently been shown to present similar pharmacological sensitivity to blocker compounds, such as terfenadine, as observed in hERG (*hKCNH2*) channels (Hull et al., 2019). Further developments to incorporate high-throughput zebrafish larvae screens revealed that bradycardia and susceptibility to 2:1 block provided accurate detection of QT prolonging compounds with high sensitivity and specificity (Burns et al., 2005; Mittelstadt et al., 2008; Peal et al., 2011; Letamendia et al., 2012) as shown in Table 1. Some studies have also used genetic models that prolong (*breakdance*, *bre*) or abbreviate (*reggae*, *reg*) the APD to demonstrate the effect of QT prolonging compounds in zebrafish larvae (Hassel et al., 2008; Milan et al., 2009; Peal et al., 2011) (see section “Zebrafish as a Model of Inherited Long-QT Syndrome” for descriptions of *breakdance* and *reggae*).

To gain mechanistic insight beyond heart rate changes, several groups developed techniques to test the impact of compounds on APD and/ECG in zebrafish larvae (see Table 1). Measurements of membrane voltage from explanted paced embryonic hearts showed that zERG block by 100 nM terfenadine prolonged the APD by 58% and resulted in 2:1 block as a result, such that only every other stimulus elicited an action potential (Arnaout et al., 2007). Using optical mapping of a fluorescent dye, Milan et al. (2009), measured the effects of a range of drugs on APD in wild-type and zERG non-trafficking variant, *breakdance*, 2 dpf zebrafish. Homozygous *breakdance* zebrafish presented marked prolongation of the APD, action potential triangulation, 2:1 block, and spontaneous EAD formation, highlighting the importance of I_*Kr*_ repolarizing current in zebrafish cardiac function. Heterozygous *breakdance* larvae presented more muted APD prolongation, but this was greatly exaggerated by the application of the hERG blocker, dofetilide, or ATX-II, which interferes with Na^+^ channel inactivation. Dofetilide alone also produced 2:1 block at higher concentrations. Following this, Peal et al. (2011), screened 1200 compounds in *breakdance* larvae using optical mapping to detect APD changes in search of compounds that might reverse the prolonged APD phenotype. They discovered two compounds that rescued wild-type-like APD in *breakdance* larvae, suggesting novel pathways for restoration of repolarization associated with hERG dysfunction. More recently, light-sheet imaging of a membrane dye in whole 14 dpf zebrafish hearts showed that E-4031 (a hERG blocker) application increases APD and induces the occurrence of frequent EAD formation (∼1 every s) (Sacconi et al., 2020). These studies demonstrate the applicability of zebrafish larvae to model acquired LQTS and to conduct toxicological screening.

### Electrical Instability and Beat-to-Beat Variability as Markers of LQTS in Zebrafish Hearts

In adult zebrafish hearts, hERG blockers prolong the APD and increase the QT interval (Milan et al., 2009; Tsai et al., 2011; Genge et al., 2016; Hull et al., 2019) while hERG activator compounds reduce APD (Hull et al., 2019; Shi et al., 2020; see Table 1). Studies using adult zebrafish hearts also permit more detailed study of markers of cardiac arrhythmogenicity, such as electrical instability and beat-to-beat variability, which is an important consideration given that although APD and QT prolongation provide a substrate for the initiation of TdP, there is evidence that QT prolongation alone correlates poorly with TdP (Mattioni et al., 1989; Shimizu et al., 1995; Fossa et al., 2002; Fenichel et al., 2004; Belardinelli et al., 2006). Frommeyer et al. (2011), suggested that the changes in AP morphology may better explain the antiarrhythmic potential of compounds. Indeed, triangulation of the action potential (often quantified as APD_90_:APD_30_), which represents prolongation of late phase repolarization or abbreviation of earlier phases, is associated with development of TdP (Hondeghem et al., 2001) as a result of increased risk for the generation of EADs and triggered activity (Frommeyer et al., 2011) and an increased temporal dispersion of repolarization which promotes re-excitation by current flow (Hondeghem et al., 2001). In adult zebrafish hearts, hERG activator compounds reduce APD with a selective effect on late phase repolarization resulting in reduced triangulation (Hull et al., 2019; Shi et al., 2020), indicating that zebrafish may be suited to screening for therapeutic compounds to ameliorate hERG channel loss of function.

Adult zebrafish hearts have also been used to study the effect of hERG activator compounds in more complex dynamic adaptation of APD to understand their antiarrhythmic potential (Shi et al., 2020). Dynamic instability of the membrane voltage is a precursor to fibrillation that arises from changes in pacing rate and can be used as a surrogate parameter in the assessment of TdP risk (Koller et al., 1995). The electrical restitution curve (ERC) provides a measure of APD stability by describing APD changes as a function of the diastolic interval (DI), the time from the end of the action potential to the activation of the following action potential (Bass, 1975; Franz, 2003). Decreasing the DI reduces the APD in the subsequent beat as a result of incomplete recovery of ion channels (Garfinkel et al., 2000; Franz, 2003; Ng et al., 2007). Changes in the shape of the ERC, in particular the maximum slope of the curve, suggest that this relationship is a promising surrogate parameter for evaluating TdP risk (Koller et al., 1995; Garfinkel et al., 2000; Franz, 2003; Ng et al., 2007). The maximum slope of the ERC measured using optical mapping of action potentials in isolated adult zebrafish hearts was increased in the presence of the hERG activator compound, RPR260243 (Shi et al., 2020), suggesting that the activator compound improved dynamic APD adaptation during acute changes in beating frequency (Franz, 2003). The increased slope of the zebrafish ERC with RPR260243 was associated with reduced beat-to-beat variability of heart rate and APD in an acquired LQTS model using dofetilide block (Shi et al., 2020) suggesting antiarrhythmic potential. Further studies using dynamic protocols to measure the ERC (using trains of stimulations with progressively shorter basic cycle lengths), which improve congruence between the observed development of alternans at short DIs and the slope of the ERC (Koller et al., 1998), promise to further enhance the predictive and translational power of ERC measurements in zebrafish hearts.

The RPR260243 hERG activator compound was also shown to increase the post-refractory repolarization period (PRRP) in zebrafish hearts (Shi et al., 2020), which has been hypothesized to be antiarrhythmic by reducing VT inducibility (Garfinkel et al., 2000; Franz, 2003; Franz et al., 2014). The PRRP is the delay in the onset of electrical restitution beyond the full repolarization of the previous action potential (Franz, 2003), i.e., it describes the phenomenon in which extrastimuli can only generate action potentials once the previous action potential has fully repolarized preventing encroachment. A longer PRRP may be considered antiarrhythmic, since the action of suppressing early premature responses to extrastimuli and allowing a more rapid normalization of APD and conduction velocity effectively narrows the window of partial refractoriness, a substrate for the generation and maintenance of VF (Franz et al., 2014). Several studies in animals and humans demonstrate the antiarrhythmic benefits of a lengthened PRRP (Koller et al., 1995; Franz, 2003; Fedorov et al., 2008) suggesting that the effect of hERG activators to prolong the PRRP in zebrafish hearts protects against TdP induction and demonstrates antiarrhythmic potential for the treatment of LQTS. Future studies using measures of electrical instability and APD rate adaptation as surrogate markers of arrhythmogenicity will benefit from correlating findings with the propensity toward EAD formation, since this is a robust biomarker. It is however interesting to note the scarcity of reported EAD events in studies of adult zebrafish hearts. Further studies aimed at characterizing the conditions required for EAD induction and triggered activity in adult zebrafish hearts would advance the use of EAD propensity as a biomarker alongside measures of electrical instability described here.

## Zebrafish as a Model of Inherited Long-QT Syndrome

Inherited LQTS2 in humans results from dysfunctional variants in the *KCNH2* gene, which encodes the hERG channel. In zebrafish, multiple variants have been identified as possible orthologs to *KCNH2*, with *zkcnh2* initially identified as the primary ortholog. However, screening for all possible zebrafish homologous sequences of *zkcnh2*, Leong et al. (2010), demonstrated the presence of a second variant, *zkcnh6a*, which paired more closely as the ortholog to *KCNH2* in humans. Tissue-specific RNA extraction and qPCR analyses have confirmed that *zkcnh6a* is the ortholog of human *KCNH2* (Langheinrich et al., 2003; Scholz et al., 2009; Leong et al., 2010; Vornanen and Hassinen, 2016; Hull et al., 2019), and the biophysical function and pharmacological properties of *zkcnh6a* channels are similar to those of human *KCNH2* (Scholz et al., 2009; Hull et al., 2019). Currents carried by *zkcnh6a* zERG channels produce the zebrafish cardiac current, I_*Kr*_, which is the predominant repolarizing driving force in the zebrafish heart (Nemtsas et al., 2010).

Performing a screen for mutations affecting zebrafish developmental, Chen et al. (1996), discovered a zERG variant associated with aberrant electrical properties. The *breakdance* mutant resulted from an I59S mutation in the N-terminal region of zERG channels and causes 2:1 atrioventricular block in zebrafish hearts (Chen et al., 1996; Milan et al., 2009; Peal et al., 2011). The cause of the A-V dissociation was subsequently show to arise from markedly delayed ventricular repolarization, which prolonged the ventricular APD such that only every other atrial depolarization resulted in ventricular capture (Milan et al., 2009; see Table 2). These findings are consistent with clinical observations in pediatric cases of LQTS, where 2:1 block is sometimes observed (Motoike et al., 2000; Chang et al., 2004; Lee et al., 2006). Kopp et al. (2005), showed that 2:1 block in *breakdance* hearts was dependent on both temperature and development, demonstrating conversion to 1:1 rhythm at lower environmental temperatures, and beyond 4 dpf. Zebrafish homozygous for the *breakdance* variant also presented markers of arrhythmogenicity, such as action potential triangulation and the presence of spontaneous EADs (Milan et al., 2009). This phenotype is consistent with LQTS in humans and the confirmation that the *breakdance* mutation is located within the zERG K^+^ channel demonstrates the potential for zebrafish to model arrhythmogenic cardiac diseases.

**TABLE 2:** Effects of inherited mutations or targeted disruption of the zERG gene on cardiac electrical activity.

Variant	Heart rate	A-V dissociation	APD	QT	Notes and other markers of arrhythmogenicity
*zkcnh2* I59S (*breakdance*) (V59 in hERG)					Non-trafficking variant.
Homozygous	85 ± 2 bpm^10^	2:1 block^3,4,7,10^	Increased from 225 ± 21 to		Triangulation and spontaneous EADs^4^
Heterozygous			615 ± 66^4^		Temperature and developmental stage dependence^10^
			Increased from 290 ± 85 to 376 ± 66^7^		Switching between 2:1 and 1:1 rhythm^10^
			Increased from 226 ± 21 to 258 ± 16^4^		
*zkcnh2* I462R (I500R in hERG)					
Homozygous		Silent ventricle^1^			
Heterozygous		2:1 block^1^			
*zkcnh2* M521K (M554K in hERG)					
Homozygous		Silent ventricle^1^	Increased from 330 ± 12 to	Increased from 416 ± 8 to	
Heterozygous		2:1 block^1^	476 ± 35 ms^1^	469 ± 25 ms^1^	
Antisense MO knockdown of *zkcnh2*	Bradycardia^2,5^	2:1 block^2,6^	APD_90_ increased from 275 ± 10 to 544 ± 15 ms^6^		MO at high concentrations produced fibrillation^2^ Depolarized resting potential and ventricular systole also observed^6^
Injection of RNA coding 40 LQTS-associated mutations and 10 non-disease causing hERG SNPs following antisense MO knockdown of *zkcnh2*		Suppression of 2:1 block in 9/10 SNPs 39/40 mutations sustained 2:1 block^6^			
*zkcnh2* antisense MO + hERG N470D (N432D in zERG) RNA			APD_90_ increased to >500 ms^6^		Function of equivalent hERG variant: no current at 37°C; non-trafficking.^11,12^
*zkcnh2* antisense MO + hERG A614V (A586V in zERG) RNA			APD_90_ increased to >500 ms^6^		Function of equivalent hERG variant: no current at 37°C; non-trafficking.^13^
*zkcnh2* antisense MO + hERG A1116V (equivalent residue position in zERG is uncertain) RNA			APD_90_ increased to >500 ms^6^		Function of equivalent hERG variant: mild QTc prolongation, reduced hERG current density when combined with K897T.^14^
*zkcnh2* L499P (*reggae*) (L532P in hERG)			APD_90_ decreased by 19%*^8^		Function of equivalent hERG variant: increased current due to right-shifted
					voltage-dependence of inactivation (reduced inactivation).^15^
					Intermittent cardiac arrest, sinus exit block.^8^
					Antisense MO knockdown of zERG suppressed reg phenotype in 48% of larvae^8^
					Cardiac function was normal in intercrossed heterozygous *reg* and *bre* larvae^8^
GFP expressed at the *zkcnh6a* locus					
Heterozygous *zkcnh6a*^*GFP/+*^		Silent ventricle^9^			GFP expressed exclusively in the heart. Larvae are viable^9^
Homozygous *zkcnh6a*^*GFP/GFP*^					Cardiac edema^9^
Conditional *zkcnh6a*^*loxP*^		Silent ventricle^9^			Cardiac edema. Cre recombinase induces excision of *zkcnh6a* exon 6^9^.

*In silico action potential model predictions. Data from Arnaout et al., 2007^1^; Langheinrich et al., 2003^2^; Chen et al., 1996^3^; Milan et al., 2009^4^, 2003^5^; Jou et al., 2013^6^; Peal et al., 2011^7^; Hassel et al., 2008^8^; Hoshijima et al., 2016^9^; Kopp et al., 2005^10^, Zhou et al., 1999^11^; Gong et al., 2004^12^; Sakaguchi et al., 2008^13^; Crotti et al., 2005^14^; Zhang et al., 2011^15^.

The utility of zebrafish as a model for inherited LQTS2 was more directly demonstrated by the identification of *zkcnh2* (likely actually *zkcnh6a*) (Leong et al., 2010) mutations, I462R (I500R in hERG) and M521K (M554K in hERG), which caused dysfunction (likely causing non-trafficking since these variants are linked with *breakdance* via complementation testing, although this has not been tested directly) of zERG channels resulting in a corresponding loss of I_*K*__*r*_ and 2:1 block when inherited heterozygously, and a silent ventricle phenotype when inherited homozygously (Arnaout et al., 2007). Recordings of action potentials from M521K (M554K in hERG) mutant zebrafish hearts revealed that the phenotype was caused by a significantly prolonged ventricular APD (Arnaout et al., 2007; see Table 2). These findings demonstrated a clear link between zERG dysfunction and cardiac phenotypes that could be readily observed and quantified in 48–72 hpf zebrafish enabling rapid detection. Other studies using antisense morpholino (MO) knockdown of zERG function showed that targeted disruption of zERG resulted in bradycardia, 2:1 block, and a prolonged APD in zebrafish embryos (Langheinrich et al., 2003; Milan et al., 2003; Jou et al., 2013). Using this as a platform, Jou et al. (2013), developed an *in vivo* cardiac assay in zebrafish embryos to screen for benign or disease-causing variants. Following MO knockdown of zERG and generation of 2:1 block or silent ventricle phenotype, injection of WT hERG RNA restored a WT-like phenotype in >50% of embryos or reduced the severity of the phenotype (i.e., 2:1 rather than silent ventricle). Similar recovery was observed with 9 of 10 non-disease causing SNPs, but not with 39 of 40 LQTS2-associated mutations, from which APD was measured in some and shown to be significantly prolonged (Jou et al., 2013; see Table 2). Furthermore, the study demonstrated a clinical phenomenon, wherein a dysfunctional variant co-expressed with a SNP was capable of reducing the phenotypic severity, showing that this assay has the potential to provide useful information for practical clinical applications. Beyond testing known LQTS-causing mutations, or screening effects of mutations found in humans, zebrafish have also been used to discover and elucidate effects of novel mutations that affect cardiac repolarization. Milan et al. (2009), used dofetilide block of zERG channels to induce 2:1 block in zebrafish embryos and rescue from or exaggeration of this phenotype was used to screen for novel gene mutations affecting repolarization. Using this approach, the authors identified 15 novel mutations involved in cardiac repolarization.

On the other end of QT-related arrhythmia spectrum, the *reggae* mutation identified in the zebrafish zERG gene causes a short QT syndrome (SQTS). Hassel et al. (2008), identified a missense mutation, L499P (L532P in hERG), which abbreviated the APD and resulted in intermittent cardiac arrest and sinus exit block. Injection of antisense MO restored the WT phenotype in around half of the *reggae* embryos consistent with the idea that the cardiac phenotype resulted from zERG gain-of-function. Furthermore, crossing of *reggae* individuals with *breakdance* individuals (in which repolarization is delayed) produced offspring with no cardiac abnormalities suggesting that the two mutations complemented one another. These studies were pioneering in their demonstration of zebrafish to model inherited LQTS. More recent developments in genetic engineering approaches promise to further unleash the potential of this model species. Below, we discuss future opportunities and challenges using precise gene-editing to create zebrafish models of inherited LQTS.

## Precise Gene-Editing Approaches in Zebrafish to Model LQTS

Precise gene editing technologies are providing one of the most rapidly evolving tools in the repertoire of genetic manipulation, and they promise to greatly influence the utility of zebrafish to model LQTS and other inherited cardiac electrical diseases. Hoshijima et al. (2016), demonstrated two novel approaches for genetic targeting and manipulation of the *zkcnh6a* gene, which provide advanced tools to model and create LQTS in zebrafish. Using transcription activator-like effector nucleases (TALENs) to target double stranded breaks in the genomic DNA immediately after the *zkcnh6a* start codon, and providing a modified synthetic DNA template for repair, which contained the coding sequence for GFP, these authors first created zebrafish lines expressing the GFP reporter gene in lieu of *zkcnh6a*. Both homozygous (*zkcnh6a*^*GFP/GFP*^) and heterozygous (*zkcnh6a*^*GFP/+*^) were generated from this approach. *zkcnh6a*^*GFP/GFP*^ embryos, homozygous for the GFP knock-in in place of *zkcnh6a*, i.e., *zkcnh6a* null, presented contractile defects and pericardial edema consistent with knock-out of zERG function (Arnaout et al., 2007; Hoshijima et al., 2016). This *zkcnh6a* knockout provides further demonstration of the role of zERG channels in zebrafish cardiac electrophysiology, and furthermore, the *zkcnh6a*^*GFP/+*^ embryos provide an additional opportunity in that heterozygous embryos, being viable (although phenotypic analyses were not performed to confirm normal cardiac function), express GFP exclusively in the heart providing a locus-specific cardiac reporter. Previous fluorescent dye membrane voltage reporters (Milan et al., 2009; Peal et al., 2011; Tsai et al., 2011) and genetically encoded fluorescent reporters (Huang et al., 2003; Burns et al., 2005) had been used to visualize or measure cardiac activity, but the approach used by Hoshijima et al. (2016), can be used to detect cardiac activity and developmental or environmental influences on *zkcnh6a* gene function specifically.

In a second advancement, Hoshijima et al. (2016), developed a silent conditional editing approach to knock-in a GFP reporter gene within the *zkcnh6a* locus. Again, using TALENs, targeted double stranded breaks were introduced in the intronic sequences on either side of the *zkcnh6a* exon 6. Repair was then directed to replace exon 6 using a novel DNA template that included the WT *zkcnh6a* exon 6 alongside GFP controlled by the α-crystallin eye lens promoter, all of which was flanked by loxP sites. Analyses revealed the introduction of the floxed allele and green eyes in 70% of zebrafish embryos, which were viable and morphologically normal. Injection of *cre* recombinase mRNA into embryos expressing the floxed allele resulted in efficient excision of sequence between the loxP sites flanking the α-crystallin:GFP and *zkcn6a* exon 6 leaving embryos without the GFP eye lens reporter, and with a silent ventricle phenotype and pericardial edema that is characteristic of knockout of *zkcnh6a* (Hoshijima et al., 2016).

Developments in genetic technology, such as in the use of Clustered Regularly Interspaced Short Palindromic Repeat (CRISPR), promise to simplify and improve efficiency of precise gene editing approaches in model systems, including zebrafish (Chang et al., 2013; Hwang et al., 2013; Varshney et al., 2015; Liu J. et al., 2017; Cornet et al., 2018). The CRISPR system is an anti-viral defense mechanism found in a variety of bacteria and archaea that has been co-opted to modify target genes within organisms (Jinek et al., 2012). CRISPR editing involves guide RNA (sgRNA) targeting of Cas-endonuclease (CRISPR associated protein) activity to a precise site within the gene of interest. Intrinsic genomic repair can occur via non-homologous end joining (NHEJ) resulting in random edits and indels, or by homology-directed repair (HDR), which can be co-opted by the provision of exogenous repair template incorporating the edit of interest. The ease of use and cost effectiveness of CRISPR has facilitated a variety of applications such as reverse genetics, improving biopharmaceutical efficiency, and allowing for the knock-out of target genes, or knock-in of point mutations within a gene of interest to generate disease models (Hruscha et al., 2013; Gupta and Musunuru, 2014; Armstrong et al., 2016). These developments hold great promise for the generation of inherited models of LQTS in zebrafish. Previous studies have shown that low success rates of precise edits via HDR (∼2–4%) (Armstrong et al., 2016; Albadri et al., 2017) using CRISPR in zebrafish can be improved by adopting a number of approaches: (1) considered design of the guide sgRNA (Doench et al., 2014; Moreno-Mateos et al., 2015; Cui et al., 2018; Michlits et al., 2020; Zhang et al., 2020); (2) delivery of sgRNA as RNA rather than DNA (Liu C. et al., 2017); (3) use of Cas9 protein instead of DNA or mRNA (Albadri et al., 2017; Zhang et al., 2018); (4) complexing Cas9 protein and sgRNA prior to injection (Burger et al., 2016); and (5) use of a plasmid-borne double-stranded repair template rather than ssODN (Irion et al., 2014). Leveraging these developments of CRISPR utility in zebrafish promises to open new possibilities for LQTS modeling in this model species. Additionally, further CRISPR-based developments, such as base-editing (Komor et al., 2016) and prime-editing (Anzalone et al., 2019) along with the development of new Cas-nucleases that utilize different recognition (Protospacer Adjacent Motif, PAM) sites (Kleinstiver et al., 2015; Hu et al., 2018) may alleviate previous limitations, such as off-target Cas9 activity and target-site restrictions. These approaches broaden the horizons and targeting range for the use of CRISPR in disease modeling in zebrafish opening the door to allow examination of the extensive array of genetic variants implicated in LQTS2 and potential underlying genetic susceptibility to acquired LQTS2, as well as other cardiac diseases.

## Challenges and Opportunities of Zebrafish as a Model of LQTS

The precision and ease with which the zebrafish genome may be modified, as described above, presents opportunities to examine clinically relevant hERG variants alongside cellular models, such as iPSC-derived cardiomyocytes (iPSC-CMs) to elucidate the phenotype of variants of unknown significance, and to inform clinical risk stratification of patients with hERG variants. Although rapidly evolving, current iPSC-CM approaches are challenged by cellular heterogeneity and immaturity, and they do not yet provide a whole organ or animal model, whereas zebrafish hearts allow for visualization of arrhythmogenicity at the tissue and whole organ level that permits examination of system-wide effects and morphological changes. There is also potential for zebrafish to be used as a whole organ companion model for the development of targeted, patient specific pharmacological approaches. The Comprehensive *in vitro* Proarrhythmia Assay (CiPA) paradigm advises inclusion of more complex approaches to arrhythmia assessment of compounds beyond heterologous expression of hERG in non-cardiac cells to include computational modeling and iPSC-derived cardiomyocyte platforms to better predict cardiotoxicity. It may be that zebrafish offer a supporting model that could be a cost-effective whole organ early toxicological screening complex model system. Indeed, recent evidence using computational approaches suggest that compound effects in zebrafish readily predict drug efficacy in human cardiomyocytes (Tveito et al., 2020).

Despite these articulated strengths that outline zebrafish as a useful model of LQTS, there are some current limitations/challenges that should be considered and that may influence interpretation or translation of findings to the human. For example, due to ancestral gene duplication events, there are alternate potential gene transcripts responsible for the zERG channel protein, and this requires knowledge and understanding of their interplay in order to carefully design targeted genetic strategies for the generation of specific LQTS-associated mutations. In addition, aside from morphological differences associated with the zebrafish two chambered heart, which might influence interpretations related to regulation of cardiac function, there are some electrophysiological differences in the zebrafish that should be considered. Although similar to hERG, zERG channel biophysical properties show some differences (Scholz et al., 2009; Hull et al., 2019) and this likely contributes to subtle differences in I_*Kr*_ current during the zebrafish action potential. Perhaps as a result of these biophysical differences, the effect of hERG activator drugs is somewhat greater on zERG than hERG channels, and combined with a prominent role for I_*Kr*_ in zebrafish hearts, this could lead to over-estimation of the effects of hERG activator compounds in zebrafish hearts and this needs to be taken into consideration. The zebrafish heart is smaller compared with the human and has reduced ventricular wall thickness, which likely contributes to altered tissue propagation and dispersion of repolarization as has been suggested (Zhao et al., 2020). With this altered electrical and physical tissue substrate, the initiation and maintenance of arrhythmias may not precisely mirror that in human hearts, and this should be taken into consideration when translating markers of arrhythmogenicity measured in zebrafish hearts. Lastly, most zebrafish experiments are conducted at 28°C, and the possible differential effects of temperature on ion channel trafficking, gating steps, and drug interactions should be considered when translating findings in zebrafish hearts to human hearts at 37°C. Notwithstanding these caveats, there remains enthusiasm for the role of the zebrafish as a translational model of both acquired and inherited LQTS that promises to continue to inform improved patient management and drug development.

## Author Contributions

KS and TC generated tables for the manuscript. KS, RV, ZP, SF, and TC contributed and drafted the manuscript together. GT provided initial input and approved the final draft. All authors approved the final draft of the manuscript.

## Conflict of Interest

The authors declare that the research was conducted in the absence of any commercial or financial relationships that could be construed as a potential conflict of interest.
